# Huge Retroperitoneal Cyst Masquerading as Ovarian Tumour: A Case Report

**DOI:** 10.3389/fsurg.2020.585411

**Published:** 2020-10-22

**Authors:** Syamim Johan, Muhammad Firdaus Hassan, Firdaus Hayati, Nornazirah Azizan, Alvin Oliver Payus, Un Hean Edwin See

**Affiliations:** ^1^Department of Surgery, Queen Elizabeth Hospital, Ministry of Health Malaysia, Kota Kinabalu, Malaysia; ^2^Department of Surgery, Faculty of Medicine and Health Sciences, Universiti Malaysia Sabah, Kota Kinabalu, Malaysia; ^3^Department of Pathobiology and Medical Diagnostic, Faculty of Medicine and Health Sciences, Universiti Malaysia Sabah, Kota Kinabalu, Malaysia; ^4^Department of Medicine, Faculty of Medicine and Health Sciences, Universiti Malaysia Sabah, Kota Kinabalu, Malaysia

**Keywords:** cystadenoma, radiography, retroperitoneal neoplasms, case report, diagnostic dilemma

## Abstract

Retroperitoneal cystic mass is a rare surgical condition that is often misdiagnosed preoperatively. Here, we report a case of a 56-year-old woman who presented with abdominal swelling for a 1-year duration, which was associated with lower abdominal pain for 6 months. Her abdominal radiograph showed a huge radiopaque lesion, and contrast-enhanced computed tomography scan of the abdomen reported it as a left ovarian serous cystadenoma causing local mass effect to the left ureter leading to mild left hydronephrosis. She underwent exploratory laparotomy and noted there was a huge retroperitoneal cystic mass. The histopathological assessment finding was consistent with a benign retroperitoneal cyst. This case report aims to share the rare case of primary retroperitoneal lesions, which can cause a diagnostic challenge preoperatively to all clinicians despite advanced achievement in medical imaging.

## Background

Retroperitoneum is the anatomical space located behind the abdominal or peritoneal cavity. It is divided into three main spaces: the anterior pararenal, perirenal, and posterior pararenal space (1). The anterior pararenal space contains the head, neck, and body of the pancreas, ascending and descending colon, and the duodenum. The structures contained within the perirenal space include the adrenal gland, kidney, ureters, and renal vessels. The posterior pararenal space, which is surrounded by the posterior leaf of the renal fascia and muscles of the posterior abdominal wall, contains no major organs and is composed primarily of fat, blood vessels, and lymphatics (2). Cystic lesions of the retroperitoneum can be subdivided into neoplastic and non-neoplastic lesions (3). Even though retroperitoneum is a rare location for primary tumors to develop, most retroperitoneal tumors are malignant. Herein, we present a 56-year-old lady who was operated on with a preoperative diagnosis of left serous ovarian cystadenoma, only to be diagnosed as retroperitoneal mass intraoperatively.

## Case Presentation

**Figure 1:**
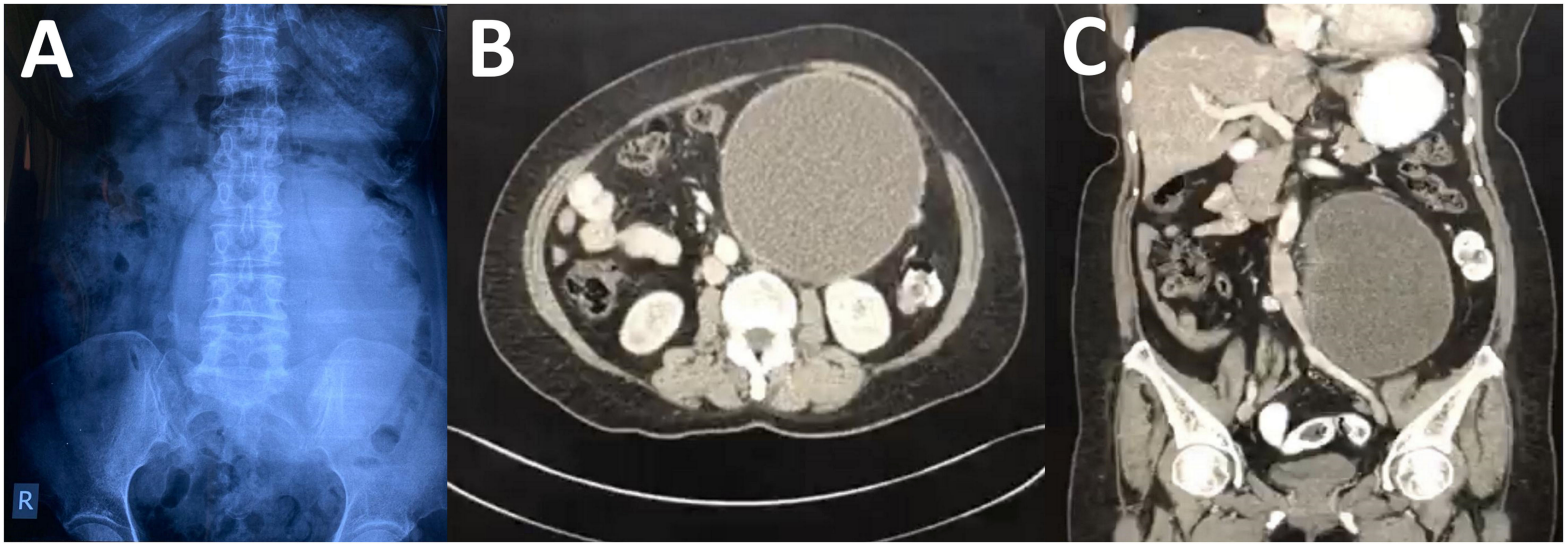
**(A)** Abdominal radiograph showing a huge oval radiopaque lesion on the left side. There was no dilated bowel visualized. **(B)** Contrast-enhanced computed tomography (CT) of the abdomen at axial phase showing a large well-defined homogenous lesion. **(C)** The homogenous lesion visualized at the coronal section.

A 56-year-old lady presented with abdominal swelling for a 1-year duration. It was associated with lower abdominal pain for 6 months, which she described as cramping in nature, localized over the lower abdomen, and resolved by taking oral analgesics. She had lost weight but was still taking orally well. She denied any history of biliary colic before. Clinically, the abdomen was soft and nontender with a mass palpable over the left lumbar area. It was measured 17 × 17 cm, mobile, and firm in consistency. There were no hepatosplenomegaly and lymph nodes palpable. The abdominal radiograph showed a huge radiopaque lesion over the left abdomen (Figure 1A). The tumor markers were within normal range, namely, serum CA 125: 6.8 (normal range: <35 U/ml), CEA: 1.3 (normal range: <50 ng/ml), and CA 19.9: <2 (normal range: <37 U/ml). Her renal profiles were also normal preoperatively; serum creatinine level ranged from 68 to 84 (normal: 50–98 μmol/L). Ultrasound of the abdomen revealed a multiloculated cyst over the left lumbar with an incomplete thick septum, and no solid component was seen. Computed tomography (CT) scan was done showing left adnexal mass with a possibility of ovarian serous cystadenoma measuring 14.5 × 14.6 × 18.2 cm with local mass effect to the ureter causing mild left hydronephrosis (Figures 1B,C). There was no other lesion seen including the gallbladder and pancreas.

**Figure 2:**
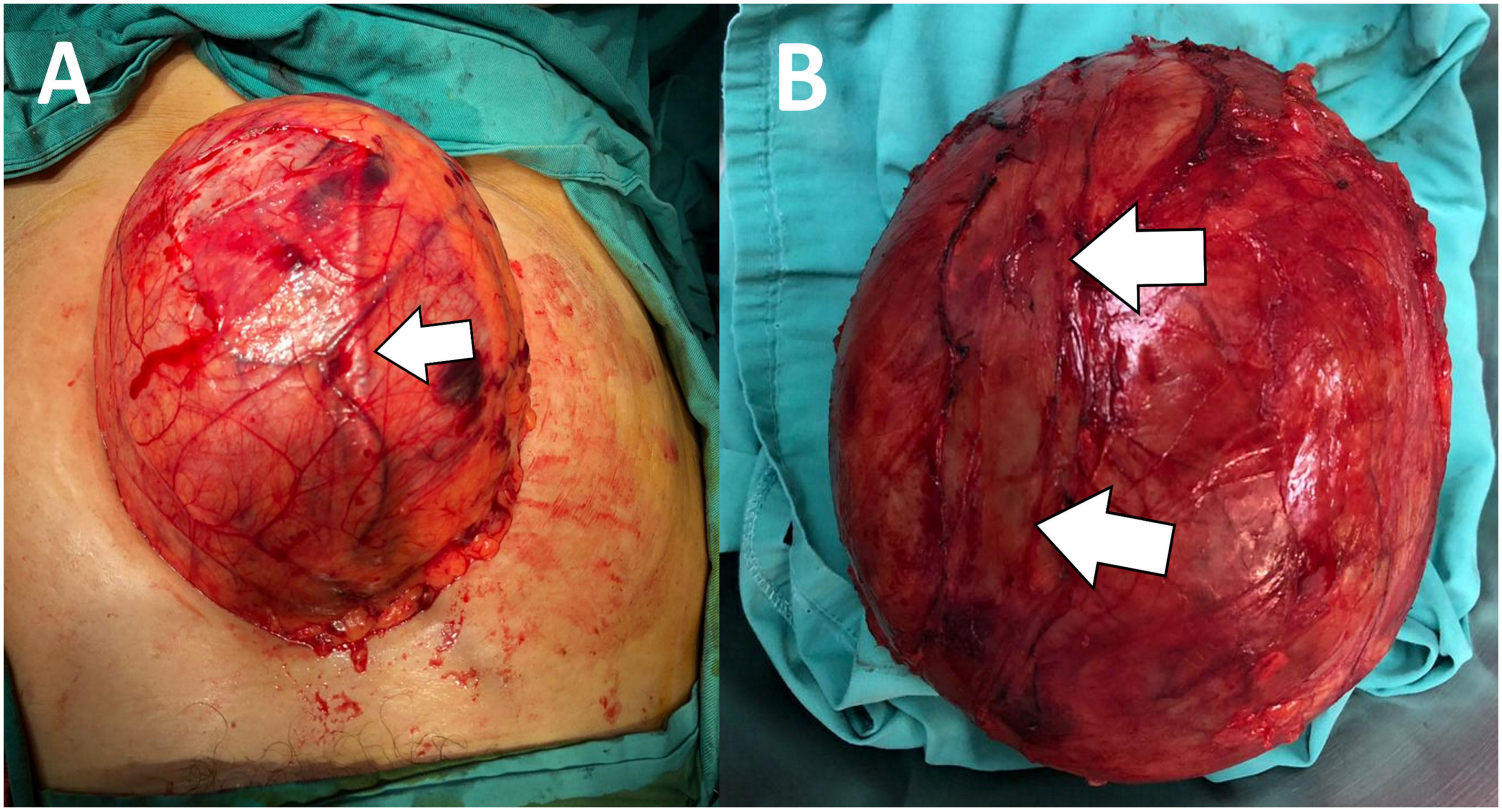
**(A)** Intraoperative picture showing a huge rounded mass with a smooth surface. There is a tubular structure likely to be the left ureter (arrow) running superficially on the mass. **(B)** Gross specimen after tumor excision showing an intact, well-encapsulated rounded lesion with preserved left ureter (previous ureter tract as shown) (arrow).

**Figure 3:**
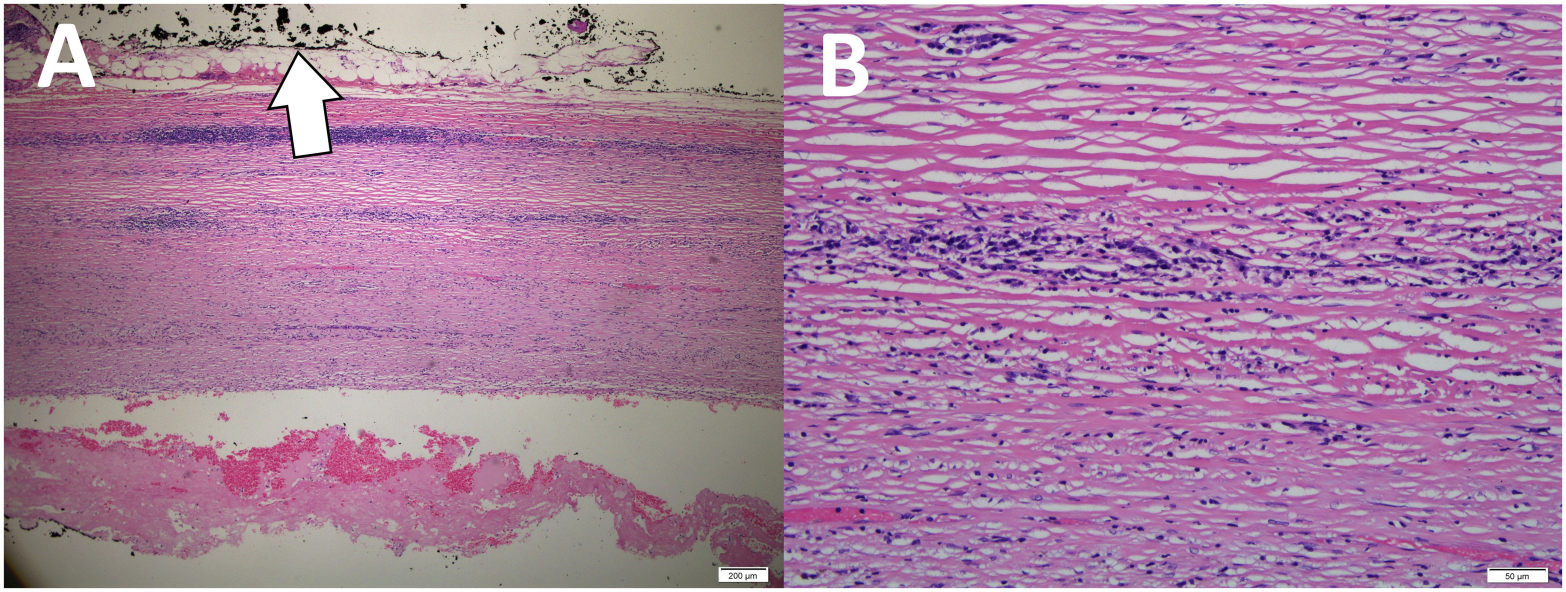
**(A)** Microscopic picture revealed sections of the cystic wall showing fibrous stroma, devoid of any epithelial lining (The outer surface of the cyst was inked black) (arrow). **(B)** Higher magnification of the cyst wall showing fibrous tissue with very mild scattered lymphocytes infiltrates.

She was well-prepared preoperatively for major surgery. She was subjected for an elective laparotomy in which left retrograde pyelogram stenting was performed. The laparotomy was carried out in a usual manner. Intraoperatively, there was a huge mass arising from the retroperitoneal space attaching to the psoas muscle, spine, and also encasing the internal iliac and left ureter (Figures 2A,B). The major organs were carefully identified and preserved. Excision of the retroperitoneal tumor was executed successfully without any intraoperative complications. She was nursed in the ward for a total of 5 days and was discharged well without any postoperative complications according to the Clavien–Dindo classification (4). The microscopic picture of the mass revealed a fibrous cyst wall devoid of epithelial lining. Focal collection of histiocytes and attached fibrin clots is seen. No malignancy was identified. The histopathological assessment finding was consistent with a benign retroperitoneal cyst (Figures 3A,B).

She was further followed up at 1-week to look for postoperative complications and renal profile, which turned up to be normal. At 3 months postoperatively, there was no recurrence identified via abdominal ultrasound. She expressed her gratitude to the operating team members who managed to help her and made her comfortable again. In view of a benign lesion, she was then discharged from our follow-up with advice after removal of the ureteric stent.

## Discussion

Retroperitoneal cystic masses are a rare surgical entity. They are usually asymptomatic, but they may grow and reach a certain size, large enough to cause symptoms especially a compression to the adjacent structures. Anterior displacement of the abdominal structures such as the aorta, colon, or kidneys may help us to identify the lesion site clinically. In this case, a huge retroperitoneal mass has given a mass effect to the left kidney causing impending renal failure. This case is unique as the external compression to the renal parenchyma is more prominent than obstruction of the ureters, which was radiologically evidenced by a merely mild hydronephrosis to occur.

There are a wide variety of differential diagnoses of retroperitoneal cystic mass. Among the potential diagnoses include neoplastic lesions (cystic lymphangioma, mucinous cystadenoma, cystic teratoma, cystic mesothelioma, Müllerian cyst, epidermoid cyst, tailgut cyst, bronchogenic cyst, cystic change in solid neoplasms, and pseudomyxoma peritoneii) and non-neoplastic lesions (pancreatic pseudocyst, non-pancreatic pseudocyst, lymphocoele, urinoma, and hematoma) (5). History and physical examination must be tailored to each aforementioned differential diagnosis especially when ruling out a neoplastic mass.

Imaging has been part of our investigation tool for the past century. Its function is undeniably important. It will help us to have more realistic and practical decision making. In a surgical point of view, it will guide us to suitably plan our steps and subsequent management in dealing with complex cases. Retroperitoneal masses can be detected incidentally or after compressive effects. Ultrasound is a non-invasive, rapid, inexpensive tool, and can easily be repeated as necessary (6). However, its role remains limited especially in the detection of intra-abdominal especially retroperitoneal pathology. The limitations include: it is an operator-dependent tool and limited in obesity, extensive bowel gases, and the presence of surgical dressing and stoma (6). CT scan is ideal for the assessment of retroperitoneal disease because it provides discrete sectional images of the organs and retroperitoneal compartments. However, in this case, we preoperatively diagnosed her having ovarian pathology with a presumed huge ovarian cyst.

Compression of the renal parenchyma by the retroperitoneal masses has been reported only once, and it was managed by insertion of the ureteral stent and systemic therapy with oral 6-mercaptopurine (7). In this case, a stent was inserted via retrograde pyelogram over the left kidney to preserve the function of the kidney. Besides, it also helps to safeguard the ureter for intraoperative identification of the organ to avoid future morbidity. Complete cystectomy (by open laparotomy or laparoscopic) remains the gold standard (3). However, in cases such as the present one, adherence to the vital structures such as the vessels or multiple viscera, partial excision with deroofing, and drainage of the cyst are appropriate. Nevertheless, the prognosis of this condition harbors a good outcome, especially in non-neoplastic types.

In conclusion, primary retroperitoneal lesions are rare and constitute a diagnostic challenge due to overlapping imaging findings. Despite preoperative imaging characteristics, intraoperative assessment is the best assessment mode. Complete cystectomy is the gold standard in managing patients with retroperitoneal mass.

## Data Availability Statement

The original contributions presented in the study are included in the article/Supplementary Material, further inquiries can be directed to the corresponding author/s.

## Informed Consent

Written informed consent was obtained from the patient for the publication of this case report.

## Author Contributions

SJ prepared and wrote this article. MFH was involved in managing the patient besides preparing the intraoperative pictures. FH wrote and revised the manuscript as well as acted as the corresponding author. NA was the anatomic pathologist who produced the histology pictures and their description. AOP wrote and reviewed this manuscript. UHES was the main surgeon and involved directly in managing the patient. All authors contributed to the article and approved the submitted version.

## Conflict of Interest

The authors declare that the research was conducted in the absence of any commercial or financial relationships that could be construed as a potential conflict of interest.
